# Knowledge and Perceptions about COVID-19 among Health Care Workers: Evidence from COVID-19 Hospitals during the Second Pandemic Wave

**DOI:** 10.3390/tropicalmed6030136

**Published:** 2021-07-19

**Authors:** Petros Ioannou, Stamatis Karakonstantis, Anna Mathioudaki, Angelos Sourris, Vasiliki Papakosta, Periklis Panagopoulos, Vasilis Petrakis, Dimitrios Papazoglou, Kostoula Arvaniti, Christina Maria Trakatelli, Evgenia Christodoulou, Garyfallia Poulakou, Konstantinos N. Syrigos, Vasiliki Rapti, Konstantinos Leontis, Dimitrios Karapiperis, Diamantis P. Kofteridis

**Affiliations:** 1 Department of Internal Medicine and Infectious Diseases, University Hospital of Heraklion, 71110 Heraklion, Greece; p.ioannou@med.uoc.gr (P.I.); stamkar2003@gmail.com (S.K.); mathiouanna94@gmail.com (A.M.); angelosourris@gmail.com (A.S.); vasopapacosta@gmail.com (V.P.); 2 Department of Infectious Diseases, Second Department of Internal Medicine, University Hospital of Alexandroupoli, 68100 Alexandroupoli, Greece; ppanago@med.duth.gr (P.P.); vasilispetrakis1994@gmail.com (V.P.); dpapazog@med.duth.gr (D.P.); 3 Infection Control Unit, COVID-19 Coordinating Team, General Hospital Papageorgiou, 56403 Thessaloniki, Greece; arvanitik@hotmail.com (K.A.); cmtrakatelli@gmail.com (C.M.T.); eugenia1381@yahoo.gr (E.C.); 4 Third Department of Medicine, Thoracic Diseases General Hospital Sotiria, 11527 Athens, Greece; gpoulakou@gmail.com (G.P.); ksyrigos@med.uoa.gr (K.N.S.); vassiarapti@gmail.com (V.R.); kostas.leontis@hotmail.com (K.L.); 5 Department of Infectious Diseases, 424 General Military Teaching Hospital, 56429 Thessaloniki, Greece; dimkarapip@yahoo.gr

**Keywords:** COVID-19, SARS-CoV-2, healthcare workers, attitudes, perceptions, knowledge

## Abstract

Health care workers (HCWs) face a higher risk of infection, since they work at the front line of COVID-19 patients’ management. Misinterpretations of current scientific evidence among HCWs may impact the delivery of appropriate care to COVID-19 patients and increase the risk of SARS-CoV-2 transmission in the hospital setting. Moreover, knowledge may affect HCWs perceptions depending on their broad beliefs and past experiences. The aim of this study was to explore the knowledge and perceptions of HCWs regarding COVID-19 issues during the second wave of the pandemic. A cross-sectional survey, involving a printed questionnaire, was conducted from 21 October 2020 to 31 January 2021 in four tertiary care hospitals located at four distant geographical regions in Greece. In total, 294 HCWs participated in this study. The majority of HCWs provided precise responses regarding general knowledge, perceptions, and practices concerning the COVID-19 pandemic. However, responses on hand hygiene and antimicrobial use in HCWs with COVID-19 were mistaken. This study reveals a certain degree of misconceptions and knowledge gaps in HCWs everyday practice, especially regarding hand hygiene and antimicrobial use in COVID-19 patients.

## 1. Introduction

Coronavirus-disease-19 (COVID-19), the disease caused by severe acute respiratory syndrome coronavirus-2 (SARS-CoV-2) has evolved into a pandemic with tremendous effects on public health, world economy, and quality of life of every individual [1]. The disease spreads from human to human primarily through droplet and direct contact and has an incubation period of 2 to 14 days [2]. Health care workers (HCWs) are in the front line of COVID-19 patients’ management, thus, they are facing constantly higher risk of infection than the wider community [3]. On the other hand, COVID-19 has affected quality of life, psychological condition, and training of HCWs, as shown in recent studies [4,5,6,7]. Several recommendations have been published from national and international societies, such as the World Health Organization (WHO) regarding the prevention and control of COVID-19 for HCWs [8]. However, misunderstandings among HCWs may negatively impact the delivery of appropriate care to COVID-19 patients and increase the risk of transmission of the virus. Additionally, knowledge gaps may affect the perceptions of HCWs, certainly depending on their beliefs and past experiences [9,10]. Except of few studies performed during the first wave of the pandemic, the level and quality of knowledge and the global perceptions of HCWs regarding COVID-19 have not been extensively studied [11].

The aim of the study was to explore the knowledge and perceptions of HCWs in COVID-19 tertiary hospitals of distal geographical regions in Greece regarding COVID-19 issues during the second wave of the pandemic. In addition, we aimed to explore the beliefs and practices of HCWs concerning personal protection equipment matters.

## 2. Materials and Methods

### 2.1. Study Design

This is a cross-sectional survey conducted from 21 October 2020 to 31 January 2021 in four tertiary care COVID-19 hospitals in four metropolitan areas in Greece. All HCWs were eligible to participate.

A printed questionnaire was developed by a team of infectious disease specialists, fellows, and internists. It consisted of 36 items, including close-ended, multiple choice, and Likert-scale questions, divided as follows: 6 on demographics and practice-related information, 4 regarding knowledge on COVID-19, 7 regarding personal beliefs on COVID-19, 7 regarding infection control measures, 3 regarding daily practice for COVID-19 patients and issues, 2 regarding COVID-19 vaccination, and 7 regarding medical evidence on COVID-19. The questionnaire is available as Supplementary Material.

### 2.2. Participation and Ethical Approval

Participation was voluntary, anonymous, and without compensation. Invitation to participate was through direct contact with a study investigator. Informed consent was distributed concomitantly with the questionnaire. The study was approved by the Ethical Committee of the University Hospital of Heraklion. 

### 2.3. Statistics

Descriptive statistics were performed with GraphPad Prism 6.0 (GraphPad Software, Inc., San Diego, CA, USA). Qualitative data were presented as counts and percentages. Continuous variables (age) were initially assessed for normality with the D’Agostino and Pearson omnibus normality test and were then presented as means with standard deviation, as they were normally distributed. Statistical analysis of quantitative data was performed through contingency analysis with chi-square test, while, a *p* < 0.05 was considered to be statistically significant.

## 3. Results

In total, 294 HCWs participated in this study. Among them, 164 (55.8%) were nurses, 114 (38.8%) medical doctors (MDs), 14 (4.8%) paramedical staff, 1 (0.3%) employee of the technical service, and 1 (0.3%) participant did not report his profession. Median age was 42 years (interquartile range: 22 to 66 years), and 103 (35%) of the participants were male. Participants’ characteristics are shown in Table 1. The questionnaire used in this study is shown in Document S1.

**Figure 1:**
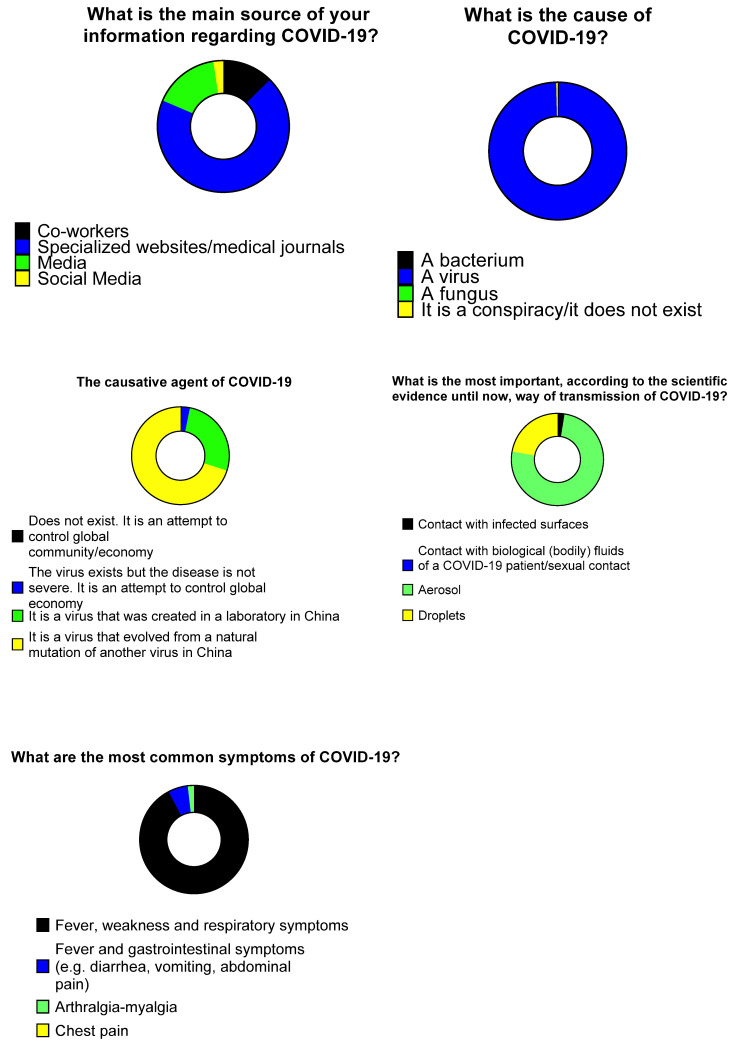
Knowledge of healthcare workers on COVID-19.

In terms of the source of participants’ information, 171 (69%) responded that the main information sources were academic journals and specialized COVID-19 websites, while 40 (16.1%) stated that their main source of information were the media. When asked about the causative agent of COVID-19, 291 (99.3%) participants stated that it is a virus. Regarding the origin of the causative agent of COVID-19, 199 (70.1%) stated it is a virus that occurred as a result of natural mutation in China, however, 76 (26.8%) answered it is a virus that was created in a Chinese laboratory. In terms of transmission, 210 (75.3%) of the responders believe COVID-19 is transmitted through aerosol, while 62 (22.2%) believe it is transmitted through droplets. When asked about the most common symptoms of COVID-19, most HCWs (252 (92.3%)) replied the disease causes fever and respiratory symptoms (Figure 1).

**Figure 2:**
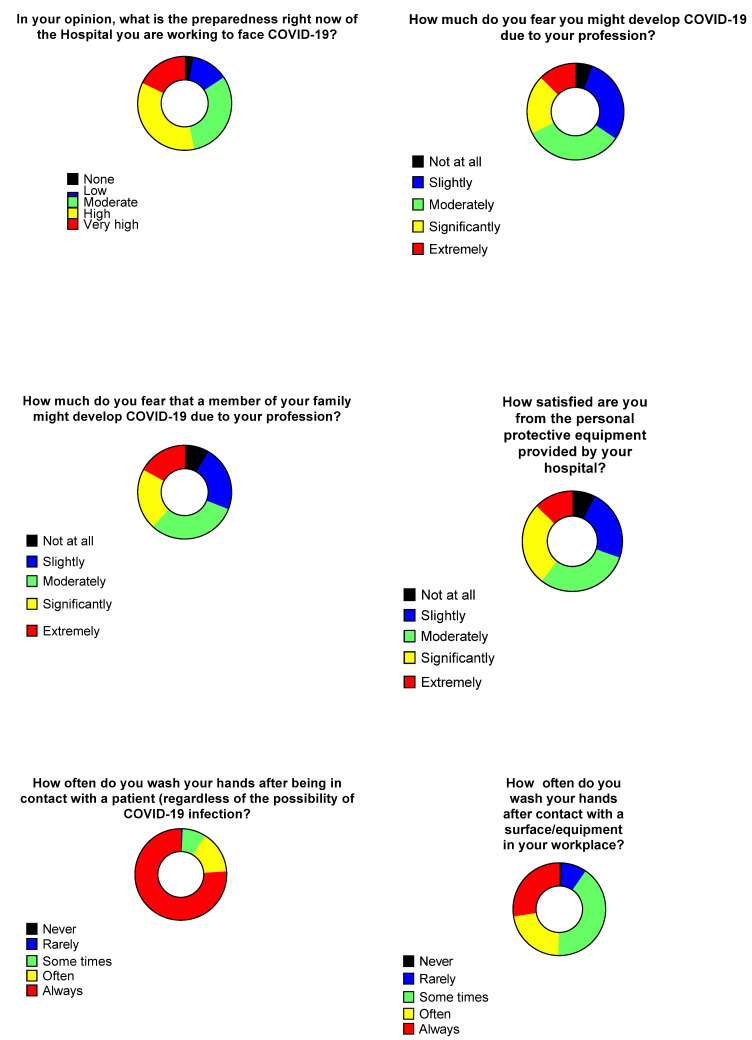
Personal perceptions regarding COVID-19 and hand-wash practices of healthcare workers.

Among the participants HCWs, 104 (35.5%) believe the preparedness of their hospital is high, 52 (17.7%) believe it is very high, while 91 (31.1%) believe it is adequate. The majority of the responders were not significantly afraid of developing COVID-19, 97 (33.1%) and 84 (28.7%) HCWs were moderately or slightly afraid, respectively, while the rates of fear of having a close relative developing COVID-19 were 91 (31%) and 66 (22.4%), respectively. The majority of the HCWs was slightly satisfied from the personal protective equipment provided from their hospital, whereas, 89 (30.3%) and 79 (26.9%) were moderately and significantly satisfied, respectively (Figure 2).

The vast majority (222, 76%) of the participants stated they always perform hand hygiene after contact with a patient, regardless of COVID-19 suspicion. On the other hand, 114 (40.7%) HCWs stated they do not always perform hand hygiene after contacting a surface or equipment in their workplace (Figure 2).

**Figure 3:**
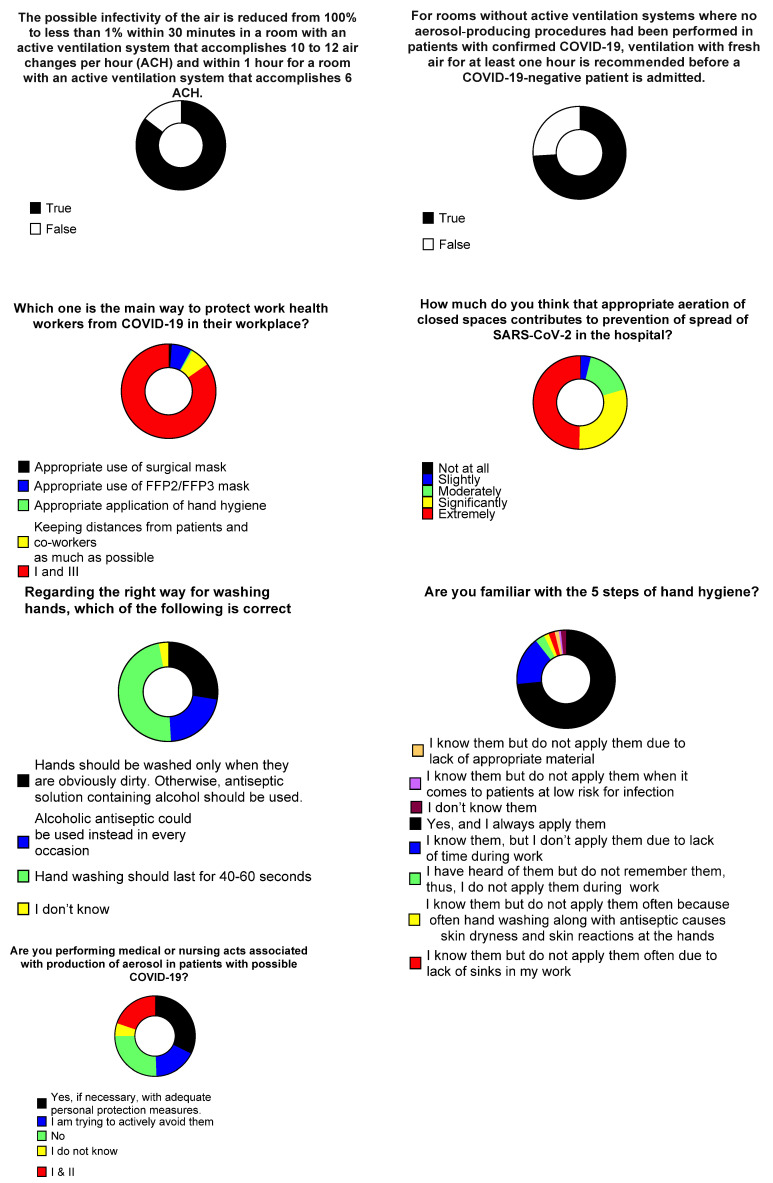
Healthcare workers’ opinions on aeration and hand-hygiene practices.

Regarding knowledge on disinfection and room ventilation guidance, 246 (85.4%) responded correctly regarding the reduction in infectivity in a room with active ventilation system, while 214 (74%) responded correctly regarding the reduction in infectivity by purely fresh air ventilation. When questioned on how much aeration of closed spaces contributes to prevention of SARS-CoV-2 hospital spread, 146 (49.7%) and 88 (29.9%) thought they contribute extremely, and significantly, respectively. When asked about the most effective method of HCWs protection against COVID-19, 149 (84.7%) answered the combination of appropriate use of surgical mask and appropriate application of hand hygiene yielded the maximum protection. Twelve responders (6.8%) believed that use of higher-protection masks (FFP2/FFP3) is most important (Figure 3).

Regarding the knowledge on hand washing 80 (47.9%) HCWs responded correctly for the appropriate practice. Notably, 46 (27.5%) responders stated that hands should only be washed when visibly dirty, and an alcoholic antiseptic solution should be used in other instances. Thirty six (21.6%) HCWs stated that hand hygiene with alcoholic antiseptics should be used in every occasion instead of hand washing. In total, 216 (73.5%) HCWs answered they know the five steps of hand hygiene and they applied them all whenever indicated, while 47 (16%) stated they know them but do not always perform them due to lack of time during their daily shifts. Female HCWs replied more often that they knew the five steps and that they always applied them compared to men. HCWs stated they were performing aerosol-producing activities in patients with possible COVID-19, 62 (32.3%) among them stated they are performing such acts with adequate preventive measures, 38 (19.8%) were performing such acts and were, also, trying to avoid them, while 49 (25.5%) were trying to avoid them most of the times (Figure 3).

When asked on the duration of isolation before returning to work in the case of COVID-19 acquisition (provided no immunosuppression and not working in a department with high-risk patients), 106 (36.6%) responded that the isolation should be 7 days, while 102 (35.2%) responded that it should be 14 days. A total of 75 (25.9%) HCWs mentioned that they were not aware, but they would consult the Hospital Infection Control Committee or the Greek National Public Health Organization. More female HCWs responded that it should be 14 days, or that they should contact the Hospital Infection Control Committee or the Greek National Public Health Organization compared to men.

**Figure 4:**
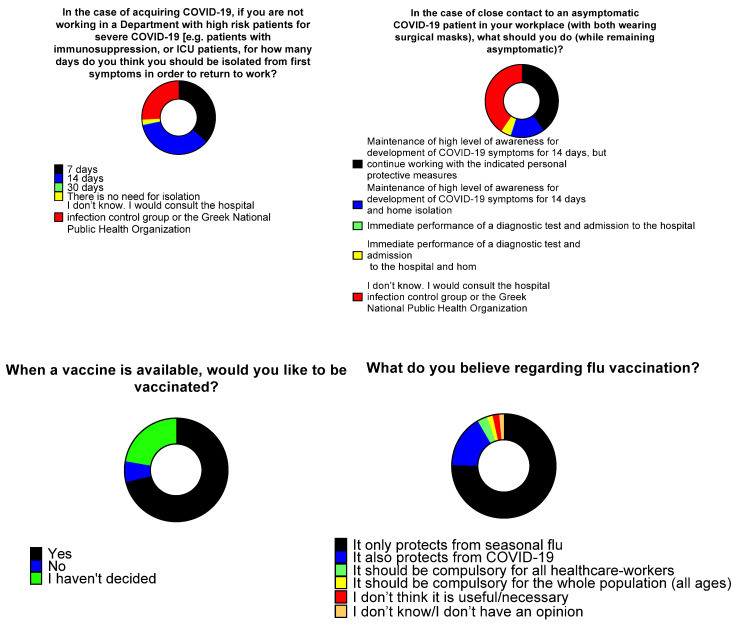
Healthcare workers’ opinions regarding isolation and vaccination.

When asked regarding their actions after close contact with an asymptomatic COVID-19 patient (both wearing surgical masks), 115 (40.5%) responded they would consult the Hospital Infection Control Committee or the Greek National Public Health Organization, 113 (39.8%) would stay at work with appropriate personal protection equipment and 14 days maintenance of high level awareness for the development of any COVID-19 symptoms and 43 (15.1%) responded they should be isolated for 14 days along with maintaining high level 14 days awareness for the development of any COVID-19 symptoms. When asked about their intention to be vaccinated against SARS-CoV-2, 208 (71.2%) responded positively, 65 (22.3%) responded that they had not decided so far, and 19 (6.5%) responded negatively. Regarding the perceptions of the HCWs on flu vaccination, 135 (47.5%) replied it only protects from flu, 31 (10.8%) replied it also protects against COVID-19, 88 (31%) replied it should be compulsory for all HCWs, and22 (7.7%) replied it should be compulsory for the whole population (Figure 4).

Among physicians, 98/114 (86%) stated their specialty, and the most common specialties were internal medicine, surgery, pulmonary medicine, and hematology in 18 (18.4%), 15 (15.3%), 11 (11.2%) and 9 (9.2%), respectively (Table S1). Overall, 50 (51.5%) physicians were attendings or consultants, and 47 (48.5%) were residents. Clinical experience was less than 5 years in 45 (44.6%), 5–10 years, and >10 years in 28 (27.7%) each. When asked about the most appropriate testing for COVID-19 diagnosis, 91 (92.9%) considered rhinopharyngeal RT-PCR as the most sensitive test, while 6 (6.1%) replied oropharyngeal RT-PCR is the most sensitive. Female HCWs were more likely to respond that oropharyngeal RT-PCR was the most sensitive. When asked about whether antimicrobials are a first line treatment for COVID-19 patients, 85 (86.7%) replied negatively and 11 (11.2%) positively. Among physicians that replied to the question whether there are specific criteria for antimicrobial prescription in COVID-19 patients, 79 (80.6%) replied positively, 9 (9.2%) negatively, and 10 (10.2%) replied they did not know. When asked regarding the most appropriate indication for starting antimicrobial treatment in COVID-19 patients, 57 (59.4%) replied all of the following are useful: procalcitonin measurement, PCR for respiratory pathogens, sputum and blood cultures, chest X-ray, and computerized tomography. Among physicians, 35 (36.1%) replied they did know what percentage of COVID-19 patients presents with co-infection by other pathogens, while 33 (34%) and 26 (28.8%), respectively, answered the percentage is 1–10% and 30–50%, respectively.

**Figure 5:**
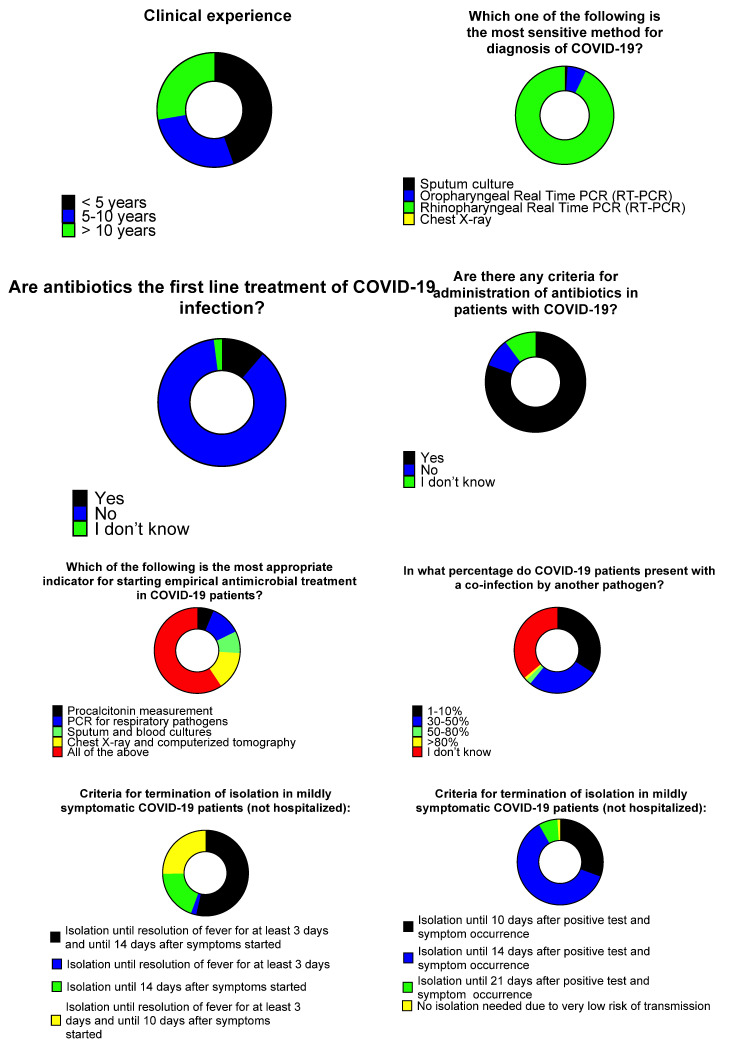
Medical doctors’ opinions and knowledge regarding antimicrobial use and isolation.

Regarding the termination of isolation for mildly symptomatic COVID-19 patients, 53 (53.5%) replied isolation should be terminated after 14 days after symptoms’ initiation (along with 3 days of defervescence), while 25 (25.3%) replied isolation should be terminated 10 days post symptoms’ initiation (along with 3 days of defervescence). When asked on the isolation period for asymptomatic patients, 60 (61.2%) replied isolation should be terminated after 14 days after the first positive test, while 30 (30.6%) said that isolation should be terminated after 10 days after the first positive test (Figure 5).

A sub-analysis of the responses was performed in order to compare the knowledge, perceptions and attitudes among physicians and non-physicians, and revealed several differences in the responses of most of the provided questions. Physicians were more likely to be informed from specialized websites and medical journals, were more likely to believe that the virus evolved from a natural mutation of another virus in China, they were more likely to respond correctly to the questions on disinfection, they were less afraid being infected by the virus in their workspace, they were less satisfied from the protective equipment of their hospital, they were more likely to wash their hands after contact with a surface or equipment in their workplace, they were more likely to know the five steps of hand hygiene, while, on the other hand, they were more knowledgeable regarding COVID-19 symptoms and isolation guidelines. Finally, physicians had a higher intention to be vaccinated when a vaccine was available. Another sub-analysis of the data in regards to the hospital revealed that HCWs from the University Hospital of Heraklion and the General Hospital Papageorgiou of Thessaloniki were slightly less afraid of being infected, or of contracting the virus, compared to their relatives.

## 4. Discussion

The present study examines the perceptions, attitudes, and practices of healthcare workers related to the COVID-19 pandemic in four major COVID-19 hospitals in Greece. The study reveals some misconceptions and knowledge gaps in everyday practice that allow for further improvement, especially in terms of hand hygiene and antimicrobial use in COVID-19 patients.

In the fight against infectious diseases, knowledge, attitudes, and practices towards these diseases can be very important, since they can affect the extent of their spread, the severity of the disease, as well as, the overall mortality rates [11,12,13]. Thus, it is important to evaluate the knowledge, perceptions, and practices of HCWs during a pandemic, in order to recognize early any misconceptions in HCWs and elaborate targeted educational initiatives and preventive interventions [14].

Female participants were twice as male participants in this study sample, while, most participants were nurses, followed by physicians. The majority of the participants had chosen scientific websites and medical journals as their main source of information regarding COVID-19, with media being the second most prevalent source. Knowledge regarding the causal pathogen, transmissibility, and COVID-19 symptoms was adequate, with most participants replying correctly to the questions. Compared to other studies evaluating the knowledge and perceptions of HCWs on COVID-19 that were performed earlier during the pandemic, our study shows superior overall knowledge of COVID-19 transmissibility and symptoms [11,15]. Similarly to our results, a recent study that was performed in Turkey, showed adequate knowledge and correct perceptions among HCWs on COVID-19 matters in the hospital setting [16].

Interestingly, a significant proportion of HCWs doubts about the preparedness of their hospital to face COVID-19 second wave, while another significant proportion is afraid about the possibility of contracting the virus and spreading the disease to their family. This is a very important finding, since anxiety and fear of acquiring COVID-19 may affect the level of provided healthcare and contribute to reduced willingness of HCWs to accept new admissions, even though their fear was associated with appropriate infection prevention practices [17]. Furthermore, fear and anxiety, in conjunction to long working hours due to increasing demands during the COVID-19 pandemic may lead to the development of mental disorders in HCWs, such as depression, suggesting that psychosocial interventions could be developed in order to support the staff [18]. We found significant compliance to infection control measures, especially in terms of hand hygiene after contact with patients, irrespective of their COVID-19 status. On the other hand, hand hygiene was not adequately performed after a contact with the patient’s surrounding environment. Misconceptions regarding the proper use of antiseptic solutions and indications for hand washing were observed in a small proportion of HCWs. This could serve as alarming issue and guide the Hospital Infection Control Committees of the participating hospitals to enhanced educational programs during the pandemic. The knowledge of HCWs regarding the hospital aeration was adequate.

Knowledge regarding isolation after exposure to a COVID-19 patient was inadequate and diverged between HCWs of the same hospital and between the participating hospitals. Interestingly, the expressed willingness for COVID-19 vaccination was around 71%, in accordance with other European studies [19,20], but was higher compared to other studies performed in healthcare professionals, that show a willingness for vaccination of about 50% [21].

Physicians, even though aware of criteria for antimicrobial prescription in COVID-19 patients, overestimated the percentage of COVID-19 patients with bacterial co-infection. As previously shown, only a minority of physicians recognized that co-infections at the time of COVID-19 diagnosis are evident in less than 10% of patients [22,23]. This could help initiate educational efforts towards antimicrobial stewardship in the pandemic era. Additionally, physicians provided diverse responses regarding the isolation guidance for patients tested positive for COVID-19, either with or without symptoms, which represents a need for focused actions towards better education, based on the local guidelines by the National Public Health Organization.

There were slight differences in the responses of male and female HCWs, with female HCWs more often stating that they know and always apply the five steps of hand hygiene, slightly more often responding that oropharyngeal RT-PCR is the most reliable means of COVID-19 diagnosis, and by supporting in a higher proportion that isolation period for HCWs exposed to SARS-CoV-2 is 14 days, or that they would consult the hospital infection control group or the Greek National Public Health Organization in the case of such exposure. This is partially in line with the literature, where female gender is associated with better hand-washing practices during the COVID-19 pandemic [24,25,26].

Responses from HCWs from different hospitals differed slightly, with the most obvious difference being the significantly, less fear noted in HCWs working in the University Hospital of Heraklion and the General Hospital Papageorgiou, Thessaloniki regarding the possibility of suffering from COVID-19, or contracting it to their relatives. This, suggests that, in general, there are no important specific local factors affecting knowledge and perceptions regarding COVID-19 infection, as most responses did not differ significantly. However, different levels of preparedness of different hospitals, factors regarding epidemiology and geographic distribution of COVID-19 cases in Greece or other factors may have influenced the perceived fear by HCWs of being infected by SARS-CoV-2. Thus, in areas with higher burden of COVID-19 cases, and lower preparedness due to financial or political reasons, perceived fear of exposure to SARS-CoV-2 may be higher. This suggests that targeted interventions to hospitals with higher burden of COVID-19 could positively affect the psychology and perceptions of HCWs, leading to improvement of their well-being, and, as a consequence, to an improvement in the healthcare provided. To that end, this study shows that centrally controlled initiatives (for example, directed from the Ministry of Health or the Greek National Public Health Organization) involving questionnaires like the one used in the present study, or audits, could help identify gaps in knowledge and practice of HCWs during the pandemic both in general, as well as more specific gaps that have to do with specific areas and hospitals. This more individualized approach could allow for interventions, such as educational activities towards the groups that require them the most, in order to improve infection control practices and increase knowledge regarding COVID-19. Interestingly, the less fear of being infected by SARS-CoV-2 in the hospitals of Heraklion and Thessaloniki does not correlate to the local trends of the pandemic, since, the second wave of the pandemic (during which, this study was performed), involved the northern part of Greece, including Thessaloniki [27,28,29].

Physicians were found in this study to be more knowledgeable regarding COVID-19, disinfection and hand-washing practices. On the other hand, they were less satisfied with the protective equipment provided by their hospital, but were also less afraid of being infected by the virus. This shows that not all HCWs share the same beliefs, or knowledge regarding COVID-19, as their education regarding healthcare in general, and COVID-19 in particular, differs. This also underlies the need for an individualized approach towards targeted interventions in order to increase awareness regarding COVID-19 and infection control practices (such as hand hygiene and disinfection). Furthermore, the increased willingness of physicians to be vaccinated suggests, as probably expected, that approval of vaccination will vary depending on the social status, the profession and other factors. This suggests that, in order to increase the approval of vaccination, HCWs and the society in general should be approached also in a structured and individualized manner, through political decisions regarding educational interventions in the workplace, media coverage, and maybe societal benefits for vaccinated individuals [30,31,32].

This study has limitations that should be mentioned. First, the exact number of persons asked to participate in the study could not have been recorded due to the nature of the questionnaire (paper-based), even though, it is estimated that the response rate was about 50% which is close to the rate noted in other studies [33,34]. Second, this is a study conducted in four tertiary COVID-19 hospitals, and the results cannot be generalized until additional data are collected from multiple hospital settings in other countries. Finally, the questionnaire reflects the perceptions, attitudes, and knowledge at the specific time period the study was conducted, as such, one cannot predict what the perceptions and attitudes of the HCWs would be in other instances, for example in times of COVID-19 vaccination generalization or in countries not experiencing a pandemic wave as the one we were experiencing in Greece at the time the study was performed.

## 5. Conclusions

To conclude, this study presented the perceptions, attitudes and practices of healthcare workers related to the COVID-19 pandemic in four COVID-19 Greek hospitals. HCWs were adequately knowledgeable regarding COVID-19 and infection control measures. Certain misconceptions and knowledge gaps in everyday practice were revealed which could promote future interventions, especially in terms of repeated education on hand hygiene and antimicrobial use in COVID-19 patients.

## Figures and Tables

**Table 1:** Participants’ characteristics. NR: not reported; SD: standard deviation.

Characteristic	Value
Gender (male), n (%)	103 (35)
Age, mean (SD)	41.8 (8.6)
HCW category	
Nurse, n (%)	164 (55.8)
Medical Doctor, n (%)	114 (38.8)
Paramedical, n (%)	14 (4.8)
Technical staff, n (%)	1 (0.3)
NR, n (%)	1 (0.3)

## Data Availability

The data presented in this study are available on request from the corresponding author.

## Supplementary Materials

The following are available online at https://www.mdpi.com/article/10.3390/tropicalmed6030136/s1, Table S1: Medical doctors’ specialties, Document S1: Questionnaire regarding knowledge, perceptions, and practice in regards to the COVID-19 pandemic.

## References

[1] Nicola M., Alsafi Z., Sohrabi C., Kerwan A., Al-Jabir A., Iosifidis C., Agha M., Agha R. (2020). The socio-economic implications of the coronavirus pandemic (COVID-19): A review. Int. J. Surg..

[2] Backer J.A., Klinkenberg D., Wallinga J. (2020). Incubation period of 2019 novel coronavirus (2019-nCoV) infections among travellers from Wuhan, China, 20–28 January 2020. Eurosurveillance.

[3] Iversen K., Bundgaard H., Hasselbalch R.B., Kristensen J.H., Nielsen P.B., Pries-Heje M.M., Knudsen A.D., Christensen C.E., Fogh K., Norsk J.B. (2020). Risk of COVID-19 in healthcare workers in Denmark: An observational cohort study. Lancet Infect. Dis..

[4] Ungureanu B.S., Vladut C., Bende F., Sandru V., Tocia C., Turcu-Stiolica R.-A., Groza A., Balan G.G., Turcu-Stiolica A. (2020). Impact of the COVID-19 Pandemic on Health-Related Quality of Life, Anxiety, and Training Among Young Gastroenterologists in Romania. Front. Psychol..

[5] Marasco G., Nardone O.M., Maida M., Boskoski I., Pastorelli L., Scaldaferri F., Italian Association of Young Gastroenterologist and Endoscopist (AGGEI) (2020). Impact of COVID-19 outbreak on clinical practice and training of young gastroenterologists: A European survey. Dig. Liver Dis..

[6] Anand S., Baishya M., Singh A., Khanna P. (2021). Effect of awake prone positioning in COVID-19 patients—A systematic review. Trends Anaesth. Crit. Care.

[7] Marcoux J.T. (2020). Training Residents during the COVID-19 Pandemic. J. Foot Ankle Surg..

[8] World Health Organization 2020 [30-10-2020]. Prevention, Identification and Management of Health Worker Infection in the Context of COVID-19. https://www.who.int/publications/i/item/10665-336265.

[9] Oppenheim B., Lidow N., Ayscue P., Saylors K., Mbala P., Kumakamba C., Kleinman M. (2019). Knowledge and beliefs about Ebola virus in a conflict-affected area: Early evidence from the North Kivu outbreak. J. Glob. Health.

[10] Vinck P., Pham P.N., Bindu K.K., Bedford J., Nilles E. (2019). Institutional trust and misinformation in the response to the 2018–19 Ebola outbreak in North Kivu, DR Congo: A population-based survey. Lancet Infect. Dis..

[11] Bhagavathula A.S., AlDhaleei W.A., Rahmani J., Mahabadi M.A., Bandari D.K. (2020). Knowledge and Perceptions of COVID-19 Among Health Care Workers: Cross-Sectional Study. JMIR Public Health Surveill..

[12] Kim J.-S., Choi J.S. (2016). Middle East respiratory syndrome-related knowledge, preventive behaviours and risk perception among nursing students during outbreak. J. Clin. Nurs..

[13] Khan M.U., Shah S., Ahmad A., Fatokun O. (2014). Knowledge and attitude of healthcare workers about middle east respiratory syndrome in multispecialty hospitals of Qassim, Saudi Arabia. BMC Public Health.

[14] Al-Shammary A.A., Hassan S.U.-N., Zahra A., Algahtani F.B.Z., Suleiman S. (2021). Role of community-based measures in adherence to self-protective behaviors during first wave of COVID-19 pandemic in Saudi Arabia. Health Promot. Perspect..

[15] Zhang M., Zhou M., Tang F., Wang Y., Nie H., Zhang L., You G. (2020). Knowledge, attitude, and practice regarding COVID-19 among healthcare workers in Henan, China. J. Hosp. Infect..

[16] Arslanca T., Fidan C., Daggez M., Dursun P. (2021). Knowledge, preventive behaviors and risk perception of the COVID-19 pandemic: A cross-sectional study in Turkish health care workers. PLoS ONE.

[17] Apisarnthanarak A., Apisarnthanarak P., Siripraparat C., Saengaram P., Leeprechanon N., Weber D.J. (2020). Impact of anxiety and fear for COVID-19 toward infection control practices among Thai healthcare workers. Infect. Control Hosp. Epidemiol..

[18] Krammer S., Augstburger R., Haeck M., Maercker A. (2020). Adjustment Disorder, Depression, Stress Symptoms, Corona Related Anxieties and Coping Strategies during the Corona Pandemic (COVID-19) in Swiss Medical Staff. Psychother. Psychosom. Med. Psychol..

[19] Verger P., Scronias D., Dauby N., Adedzi K.A., Gobert C., Bergeat M., Gagneur A., Dubé E. (2021). Attitudes of healthcare workers towards COVID-19 vaccination: A survey in France and French-speaking parts of Belgium and Canada, 2020. Eurosurveillance.

[20] Gagneux-Brunon A., Detoc M., Bruel S., Tardy B., Rozaire O., Frappe P., Botelho-Nevers E. (2021). Intention to get vaccinations against COVID-19 in French healthcare workers during the first pandemic wave: A cross-sectional survey. J. Hosp. Infect..

[21] Turcu-Stiolica A., Bogdan M., Subtirelu M.-S., Meca A.-D., Taerel A.-E., Iaru I., Kamusheva M., Petrova G. (2021). Influence of COVID-19 on Health-Related Quality of Life and the Perception of Being Vaccinated to Prevent COVID-19: An Approach for Community Pharmacists from Romania and Bulgaria. J. Clin. Med..

[22] Langford B.J., So M., Raybardhan S., Leung V., Westwood D., MacFadden D.R., Soucy J.-P.R., Daneman N. (2020). Bacterial co-infection and secondary infection in patients with COVID-19: A living rapid review and meta-analysis. Clin. Microbiol. Infect..

[23] Lansbury L., Lim B., Baskaran V., Lim W.S. (2020). Co-infections in people with COVID-19: A systematic review and meta-analysis. J. Infect..

[24] Czeisler M.É., Garcia-Williams A.G., Molinari N.-A., Gharpure R., Li Y., Barrett C.E., Robbins R., Facer-Childs E.R., Barger L.K., Czeisler C.A. (2020). Demographic Characteristics, Experiences, and Beliefs Associated with Hand Hygiene Among Adults during the COVID-19 Pandemic—United States, June 24–30, 2020. MMWR Morb. Mortal. Wkly. Rep..

[25] Guzek D., Skolmowska D., Głąbska D. (2020). Analysis of Gender-Dependent Personal Protective Behaviors in a National Sample: Polish Adolescents’ COVID-19 Experience (PLACE-19) Study. Int. J. Environ. Res. Public Health.

[26] Sax H., Uçkay I., Richet H., Allegranzi B., Pittet D. (2007). Determinants of Good Adherence to Hand Hygiene among Healthcare Workers Who Have Extensive Exposure to Hand Hygiene Campaigns. Infect. Control Hosp. Epidemiol..

[27] Nørgaard S.K., Vestergaard L.S., Nielsen J., Richter L., Schmid D., Bustos N., Braye T., Athanasiadou M., Lytras T., Denissov G. (2021). Real-time monitoring shows substantial excess all-cause mortality during second wave of COVID-19 in Europe, October to December 2020. Eurosurveillance.

[28] Siettos C., Anastassopoulou C., Tsiamis C., Vrioni G., Tsakris A. (2021). A bulletin from Greece: A health system under the pressure of the second COVID-19 wave. Pathog. Glob. Health.

[29] Post L., Culler K., Moss C.B., Murphy R.L., Achenbach C.J., Ison M.G., Resnick D., Singh L.N., White J., Boctor M.J. (2021). Surveillance of the Second Wave of COVID-19 in Europe: Longitudinal Trend Analyses. JMIR Public Health Surveill..

[30] Lang R., Benham J.L., Atabati O., Hollis A., Tombe T., Shaffer B., Burns K.K., MacKean G., Léveillé T., McCormack B. (2021). Attitudes, behaviours and barriers to public health measures for COVID-19: A survey to inform public health messaging. BMC Public Health.

[31] McKee M., Rajan S. (2021). What can we learn from Israel’s rapid roll out of COVID 19 vaccination?. Isr. J. Health Policy Res..

[32] Wake A.D. (2021). The Willingness to Receive COVID-19 Vaccine and Its Associated Factors: “Vaccination Refusal Could Prolong the War of This Pandemic”—A Systematic Review. Risk Manag. Healthc. Policy.

[33] Spernovasilis N., Ierodiakonou D., Spanias C., Mathioudaki A., Ioannou P., Petrakis E., Kofteridis D. (2021). Doctors’ Perceptions, Attitudes and Practices towards the Management of Multidrug-Resistant Organism Infections after the Implementation of an Antimicrobial Stewardship Programme during the COVID-19 Pandemic. Trop. Med. Infect. Dis..

[34] Perozziello A., Lescure F.X., Truel A., Routelous C., Vaillant L., Yazdanpanah Y., Lucet J.C., Liem-Binh L.N., Bruno M., CEFECA Study Group (2019). Prescribers’ experience and opinions on antimicrobial stewardship programmes in hospitals: A French nationwide survey. J. Antimicrob. Chemother..

