# Supplementary material for: Knowledge and Perceptions about COVID-19 among Health Care Workers: Evidence from COVID-19 Hospitals during the Second Pandemic Wave
Source: Trop Med Infect Dis. 2021 Jul 19;6(3):136. doi: 10.3390/tropicalmed6030136 (PMC8293362; doi:10.3390/tropicalmed6030136)
Supplement: Supplementary file 1 [file tropicalmed-06-00136-s001.zip › Supplementary document - Questionnaire.pdf]

Questionnaire regarding knowledge, perceptions and practice in regards to the COVID-19 pandemic

Hospital……………………….

Gender

- Male
- Female
- Other: ……………………………

Age:

Profession

- Medical Doctor
- Nurse
- Other:…………………………..

1. **What is the main source of your information regarding COVID-19?**
- Co-workers
- Specialized websites/medical journals
- Media
- Social Media

2. **What is the cause of COVID-19?**
- A bacterium
- A virus
- A fungus
- It is a conspiracy/it does not exist

3. **The causative agent of COVID-19:**
- Does not exist. It is an attempt to control global community/economy.
- The virus exists but the disease is not severe. It is an attempt to control global community/economy.
- It is a virus that was created in a laboratory in China
- It is a virus that evolved from a natural mutation of another virus in China

4. **In your opinion, what is the preparedness right now of the Hospital you are working to face COVID-19?**

- o None
- o Low
- o Moderate
- o High
- o Very high

5. **What is the <u>most important, according to the scientific evidence until now,</u> way of transmission of COVID-19?**

- o Contact with infected surfaces
- o Contact with biological (bodily) fluids of a COVID-19 patient/sexual contact
- o Aerosol
- o Droplets

6. **According to the guidelines of cleaning and disinfection of surface and clothing, the following should be used/is correct:**

- o Fresh sodium hypochlorite solution in a concentration of 0.1-0.5% (1000 ppm – 5000 ppm available chloride) for at least one minute
- o Ethanol based solution, in a concentration of 70-80%, for at least one minute
- o Clothing should be washed in the washing machine at a temperature of (60°-90^oC) using usual detergent with or without chlorine
- o I don’t know

7. **The possible infectivity of the air is reduced from 100% to less than 1% within 30 minutes in a room with an active ventilation system that accomplishes 10 to 12 air changes per hour (ACH) and within 1 hour for a room with an active ventilation system that accomplishes 6 ACH.**

- o True
- o False

8. **For rooms without active ventilation systems where no aerosol-producing procedures had been performed in patients with confirmed COVID-19, ventilation with fresh air for at least one hour is recommended before a COVID-19-negative patient is admitted.**

- True
- False

9. **Which one is the <u>main way</u> to protect work health workers from COVID-19 in their workplace?**
   I. Appropriate use of surgical mask
   II. Appropriate use of FFP2/FFP3 mask
   III. Appropriate application of hand hygiene
   IV. Keeping distances from patients and co-workers as much as possible
   V. I and III

10. **How much do you fear you might develop COVID-19 due to your profession?**
- Not at all
- Slightly
- Moderately
- Significantly
- Extremely

11. **How much do you fear that a member of your family might develop COVID-19 due to your profession?**
- Not at all
- Slightly
- Moderately
- Significantly
- Extremely

12. **How satisfied are you from the personal protective equipment provided by your hospital?**
- Not at all
- Slightly
- Moderately
- Significantly
- Extremely

13. **How often do you wash your hands after being in contact with a patient (regardless of the possibility of COVID-19 infection?**
- Never
- Rarely
- Some times

- Often
- Always

14. How often do you wash your hands after contact with a surface/equipment in your workplace?

- Never
- Rarely
- Some times
- Often
- Always

15. Regarding the right way for washing hands, which of the following is correct:

- Hands should be washed only when they are obviously dirty. Otherwise, antiseptic solution containing alcohol should be used.
- Alcoholic antiseptic could be used instead in every occasion
- Hand washing should last for 40-60 seconds.
- I don’t know

16. Are you familiar with the 5 steps of hand hygiene?

- Yes, and I always apply them
- I know them, but I don’t apply them due to lack of time during work
- I have heard of them but do not remember them, thus, I do not apply them during work
- I know them but do not apply them often because often hand washing along with antiseptic causes skin dryness and skin reactions at the hands
- I know them but do not apply them often due to lack of sinks in my work
- I know them but do not apply them due to lack of appropriate material (alcoholic antiseptic solutions, antiseptic soap, paper towels)
- I know them but do not apply them when it comes to patients at low risk for infection
- I don’t know them

17. How much do you think that appropriate aeration of closed spaces contributes to prevention of spread of SARS-CoV-2 in the hospital?

- Not at all
- Slightly

- Moderately
- Significantly
- Extremely

18. Are you performing medical or nursing acts associated with production of aerosol in patients with possible COVID-19?

- Yes, if necessary, with adequate personal protection measures.
- I am trying to actively avoid them.
- No
- I do not know
- I & II

19. What are the <u>most common symptoms</u> of COVID-19?

- Fever, weakness and respiratory symptoms
- Fever and gastrointestinal symptoms (e.g. diarrhea, vomiting, abdominal pain)
- Arthralgia-myalgia
- Chest pain

20. In the case of acquiring COVID-19, if you are not working in a Department with high-risk patients for severe COVID-19 [e.g. patients with immunosuppression, or ICU patients], for how many days do you think you should be isolated from first symptoms in order to return to work?

- 7 days
- 14 days
- 30 days
- There is no need for isolation
- I don't know. I would consult the hospital infection control group or the Greek National Public Health Organization

21. In the case of close contact to an asymptomatic COVID-19 patient in your workplace (with both wearing surgical masks), what should you do (while remaining asymptomatic)?

- Maintenance of high level of awareness for development of COVID-19 symptoms for 14 days, but continue working with the indicated personal protective measures
- Maintenance of high level of awareness for development of COVID-19 symptoms for 14 days and home isolation

- Immediate performance of a diagnostic test and admission to the hospital
- Immediate performance of a diagnostic test and admission to the hospital and home isolation for 14 days
- I don’t know. I would consult the hospital infection control group or the Greek National Public Health Organization

22. When a vaccine is available, would you like to be vaccinated?

- Yes
- No
- I haven’t decided

23. What do you believe regarding flu vaccination?

- It only protects from seasonal flu
- It also protects from COVID-19
- It should be compulsory for all healthcare-workers
- It should be compulsory for the whole population (all ages)
- I don’t think it is useful/necessary
- I don’t know/I don’t have an opinion

If you are a medical doctor, please reply to the following questions as well:

24. Medical Specialty:…………………………..

25. Position

- Attending physician / consultant
- Resident
- Rural doctor

26. Clinical experience

- < 5 years
- 5-10 years
- > 10 years

27. Which one of the following is the most sensitive method for diagnosis of COVID-19?

- Sputum culture
- Oropharyngeal Real Time PCR (RT-PCR)
- Rhinopharyngeal Real Time PCR (RT-PCR)
- Chest X-ray

28. Are antibiotics the first line treatment of COVID-19 infection?
- Yes
- No
- I don’t know

29. Are there any criteria for administration of antibiotics in patients with COVID-19?
- Yes
- No
- I don’t know

30. Which of the following is the most appropriate indicator for starting empirical antimicrobial treatment in COVID-19 patients?
- Procalcitonin measurement
- PCR for respiratory pathogens
- Sputum and blood cultures
- Chest X-ray and computerized tomography
- All of the above

31. In what percentage do COVID-19 patients present with a co-infection by another pathogen?
- 1-10%
- 30-50%
- 50-80%
- >80%
- I don’t know

32. Criteria for termination of isolation in mildly symptomatic COVID-19 patients (not hospitalized):
- Isolation until resolution of fever for at least 3 days and until 14 days after symptoms started
- Isolation until resolution of fever for at least 3 days
- Isolation until 14 days after symptoms started
- Isolation until resolution of fever for at least 3 days and until 10 days after symptoms started

33. Criteria for termination of isolation in asymptomatic COVID-19 patients:

- o Isolation until 10 days after positive test and symptom occurrence
- o Isolation until 14 days after positive test and symptom occurrence
- o Isolation until 21 days after positive test and symptom occurrence
- o No isolation needed due to very low risk of transmission
