# Supplementary material for: Knowledge and Perceptions about COVID-19 among Health Care Workers: Evidence from COVID-19 Hospitals during the Second Pandemic Wave
Source: Trop Med Infect Dis. 2021 Jul 19;6(3):136. doi: 10.3390/tropicalmed6030136 (PMC8293362; doi:10.3390/tropicalmed6030136)
Supplement: Supplementary file 1 [file tropicalmed-06-00136-s001.zip › Supplementary Table S1.pdf]

Supplementary Table S1. Medical doctors' specialties.

Specialty	N (% among responders)
Internal Medicine	18 (18.4)
General Surgery	15 (15.3)
Pulmonary Medicine	11 (11.2)
Hematology	9 (9.2)
Obstetrics – Gynecology	6 (6.1)
Nephrology	6 (6.1)
Neurology	5 (5.1)
General Medicine	4 (4.1)
Cardiology	4 (4.1)
Gastroenterology	3 (3.1)
Endocrinology	3 (3.1)
Orthopedics	3 (3.1)
Pediatrics	2 (2)
Dermatology	2 (2)

Urology	1 (1)
Nuclear Medicine	1 (1)
Microbiology & Laboratory Medicine	1 (1)
Oncology	1 (1)
Cardiac surgery	1 (1)
Neurosurgery	1 (1)
Intensive Care & Infectious Diseases	1 (1)
NR	16

NR: not reported
